# Apatinib treatment of advanced hepatocellular carcinoma with portal vein and inferior vena cava tumor thrombus: A case report

**DOI:** 10.1097/MD.0000000000014582

**Published:** 2019-02-22

**Authors:** XueGang Yang, Ge Wu, GuoHui Xu

**Affiliations:** Department of Interventional Radiology, Sichuan Cancer Hospital and Institute, Sichuan Cancer Center, School of Medicine, University of Electronic Science and Technology of China, Chengdu, Sichuan Province, China.

**Keywords:** angiogenesis, apatinib, hepatocellular carcinoma, tumor thrombus

## Abstract

**Rationale:**

Hepatocellular carcinoma (HCC) is a highly invasive cancer associated with vascular invasion. The survival of advanced HCC is very poor. In this case study, we describe the efficacy and safety of apatinib in patient with advanced HCC as the first-line therapy.

**Patient concerns:**

A 46-year-old male complained of abdominal distention and pain for half a month.

**Diagnoses:**

HCC patient with portal vein and inferior vena cava tumor thrombus.

**Interventions:**

The apatinib alone was used as first-line therapy.

**Outcomes:**

Intrahepatic tumors, portal vein, and inferior vena cava tumor thrombus were diminished. The patient achieved partial response (PR) soon after the treatment, and progression-free survival (PFS) was 12.5 months. During the entire process, the alpha-fetoprotein (AFP) continued to decrease.

**Lessons:**

Apatinib alone may be a safe and effective therapy for HCC patients with portal vein and inferior vena cava tumor thrombus. However, it is warranted further investigation in the future prospective randomized clinical studies.

## 1 Introduction

Hepatocellular carcinoma (HCC) is one of the most common malignant tumors in world.^[1]^ In China, HCC is the third leading cause of cancer death among both women and men.^[2]^ Unfortunately, it has been estimated that up to 60% to 70% of patients with HCC are diagnosed at intermediate-to-advanced stage, where hepatic resection and liver transplantation are not feasible.^[3]^ Only palliative treatment options are available for these patients.^[4]^ Sorafenib is the standard first-line treatment for advanced HCC.^[5]^ However, the treatment efficacy of it was limited and expensive.

Apatinib (Hengrui Pharmaceutical Co., Ltd, Shanghai, People's Republic of China) is a novel VEGFR-2 inhibitor that has the highest selectivity; it can block the migration and proliferation of vascular endothelial cells, reduce tumor microvessel density, and inhibit tumor growth.^[6]^ Clinical trials have proved the safety and effect of apatinib on metastatic triple negative breast cancer, advanced gastric cancer and intrahepatic cholangiocarcinoma.^[7–9]^ Apatinib is a first-generation oral antiangiogenesis drug approved in China for use as a subsequent line of treatment for advanced gastric cancer. Here we report a case using apatinib treatment of HCC with portal vein and inferior vena cava tumor thrombus in our hospital.

## 2 Case presentation

**Figure 1:**
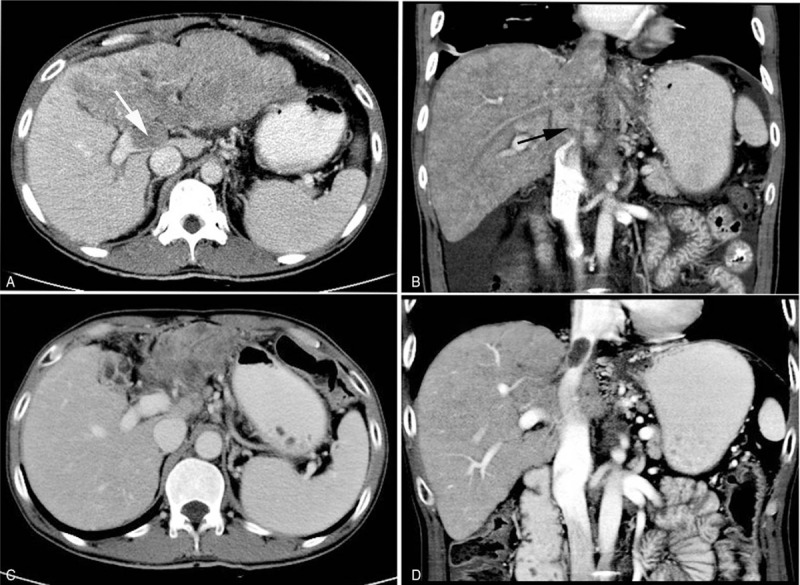
Abdomen CT images show that lesions ware located in left lobe of the liver. (A) In the venous phase, the mass is low density and irregular; the white arrow represents tumor thrombosis in portal vein. (B) The black arrow represents tumor thrombosis in inferior vena cava. (C) Intrahepatic tumors and tumor thrombosis in portal vein were diminished after oral aptinib 2 months. (D) Tumor thrombosis in inferior vena cava was diminished after 2 months.

**Figure 2:**
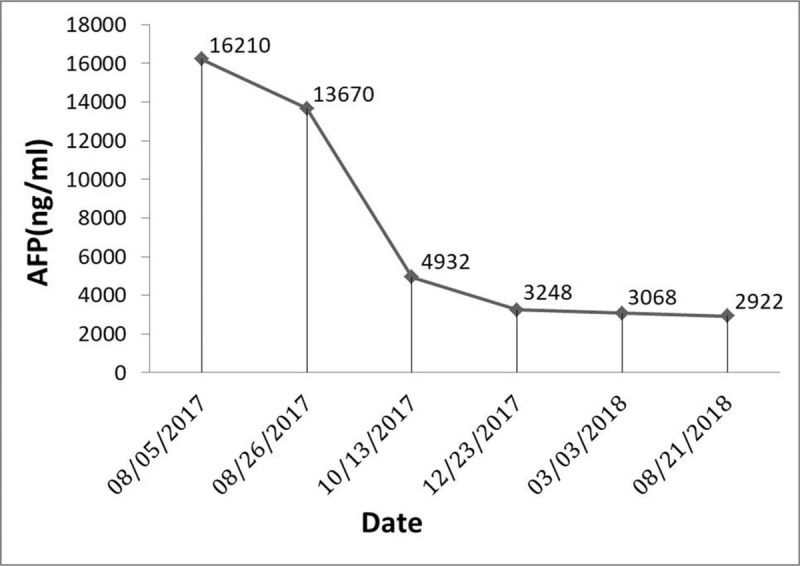
The level of serum AFP was continued to decrease during treatment.

A 46-year-old man was referred to our hospital with complains of abdominal distention and pain for half a month in August 2017. This patient had Visual Analogue Score (VAS) of 5. He had a history of chronic hepatitis B for >15 years, and HBsAg, HbeAb, and HBcAb positive, respectively. Physical examination showed abdominal bulging and both lower extremity edema. Abdominal enhancement computed tomography (CT) scan revealed multiple masses in the liver. These masses located at the left lobe of the liver, with a maximum volume of 17.6 × 7.9 cm (Fig. 1A), and portal vein tumor thrombosis, hepatic vein, and inferior vena cava tumor thrombosis were showed on CT (Fig. 1A and B). Eastern Cooperative Oncology Group (ECOG) performance score 2, Child-Pugh grade 10, and serum alpha-fetoprotein (AFP) were 16210 ng/mL. The diagnosis of HCC was carried out according to the American Association for the Study of Liver Diseases (AASLD) Practice Guideline.^[10]^ This patient lost treatment opportunities of surgery, liver transplantation, and transcatheter arterial chemoembolization (TACE). Then, he received apatinib (500 mg once daily) treatment. Symptoms of abdominal distension and both lower extremity edema diminished with 1 month and the VAS of the patient improved from 5 to 2. AFP was decreased from 16210 to 13670 ng/mL after 21 days of treatment (Fig. 2). Intrahepatic tumors, portal vein, and inferior vena cava tumor thrombus were significantly diminished after 2 months of treatment. Partial response (PR) was detected (Fig. 1C and D) after 2 months of treatment. Progression-free survival (PFS) after apatinib treatment was 12.5 months. The ECOG performance score was 1 of the patient on December 20, 2018. The patient had been followed for 16 months. The main toxicities were grade 2 hand-foot skin reaction and grade 1 hypertension, which were well controlled.

## 3 Discussion

According to the Barcelona Clinic Liver Cancer (BCLC) clinical staging system, the standard treatment for HCCs in BCLC stage C oral Sorafenib.^[11]^ Sorafenib is a multiple signaling pathways multikinase inhibitor.^[5]^ However, disease control with sorafenib is limited.^[5]^ It is necessary to look for affordable molecular targeted drug for advanced HCC. Angiogenesis is mediated by vascular endothelial growth factor (VEGF) and acts as an important role in the process of tumor growth and metastasis.^[12]^ Vascular endothelial growth factor receptor (VEGFR) family proteins are membrane receptor tyrosine kinases, including VEGFR-1, VEGFR-2, and VEGFR-3.^[13]^ VEGF-2 is closely associated with the occurrence of tumor.

Apatinib is the highest selectivity inhibitor of VEGFR-2 targeting the intracellular ATP-binding site of the receptor.^[6,14]^ In a randomized, open-label, multicenter phase II clinical study, the effects of 2 different doses of apatinib for patients with advanced HCC as the first-line therapy on the time to progression (TTP) and median OS were compared. The TTP of patients who received 850 mg once daily versus 750 mg once daily of apatinib were 4.21 months versus 3.32 months in 850 and 750 mg groups (*P* > .05), respectively. The median OS was 9.71 months versus 9.82 months, respectively.^[15]^ The drug-related adverse events included hand-foot syndrome, hypertension, and fatigue, which were well controlled. And adverse events were similar in the 2 groups. So, they recommend 750 mg once daily. Recently, apatinib also shows significant efficacy and well safety in patients with intermediate/advanced hepatocellular carcinoma. In the study, a total of 31 patients with HCC were administered oral apatinib 500 mg once daily and the median TTP was 4.8 months.^[16]^ In this case study, the patient was administered oral apatinib 500 mg daily. The drug-related adverse events were grade 2 hand-foot skin reaction and grade 1 hypertension. He has achieved a PFS of 12.5 months.

In this case, apatinib showed its effective therapeutic action and extended the life expectancy. So, we think that apatinib may be a good choice for HCC with portal vein and inferior vena cava tumor thrombus. However, it is a challenge how to select patients who are more likely to be responsive to apatinib, and further large-scale prospective studies are required to prove the effect of apatinib in HCC with portal vein and inferior vena cava tumor thrombus.

Apatinib alone as first-line treatment of a HCC patient with portal vein and inferior vena cava tumor thrombus showed an excellent antitumor effect. Intrahepatic tumors, portal vein, and inferior vena cava tumor thrombus were diminished. The patient achieved PR after treatment of 2 months, which lasted for 12.5 months. Apatinib alone may be a good choice for advanced HCC with portal vein and inferior vena cava tumor thrombus; however, further investigation in future prospective randomized clinical studies is warranted.

## Acknowledgments

The authors are grateful to the patient, who gave his informed consent for publication.

## Author contributions

**Conceptualization:** Xuegang Yang, Ge Wu.

**Data curation:** Xuegang Yang, Ge Wu.

**Formal analysis:** Xuegang Yang.

**Funding acquisition:** Xuegang Yang, Ge Wu.

**Investigation:** Xuegang Yang, Ge Wu.

**Methodology:** Xuegang Yang, Ge Wu, Guohui Xu.

**Project administration:** Xuegang Yang, Ge Wu.

**Resources:** Xuegang Yang, Ge Wu.

**Supervision:** Xuegang Yang, Ge Wu.

**Visualization:** Xuegang Yang.

**Writing – original draft:** Xuegang Yang.

**Writing – review and editing:** Xuegang Yang, Ge Wu, Guohui Xu.
